# Assessing needs for psychiatric treatment in prisoners: 2. Met and unmet need

**DOI:** 10.1007/s00127-016-1313-5

**Published:** 2016-11-22

**Authors:** Sharon Jakobowitz, Paul Bebbington, Nigel McKenzie, Rachel Iveson, Gary Duffield, Mark Kerr, Helen Killaspy

**Affiliations:** 1 Division of Psychiatry, University College London, 6th Floor, Maple House, 149 Tottenham Court Road, London, W1T 7NF UK; 2 Camden and Islington Foundation Trust, St Pancras Hospital, 4 St Pancras Way, London, NW1 OPE UK; 3 North London Forensic Service, Chase Farm Hospital, The Ridgeway, Enfield, Middlesex EN2 8JL UK; 4 School of Social Policy, Sociology and Social Research, Faculty of Social Sciences, Cornwallis North East, University of Kent at Canterbury, Canterbury, Kent CT2 7NF UK

**Keywords:** Prisoners, Psychiatric disorders, Psychosis, Needs for care, Treatment

## Abstract

**Background:**

In a companion paper, we established high levels of psychiatric morbidity in prisoners (Bebbington et al. Soc Psychiatry Psychiatr Epidemiol, 2016). In the current report, we evaluate how this morbidity translates into specific needs for treatment and the consequent implications for services. Mental health treatment needs and the extent to which they had been met were assessed in a representative sample of prisoners in a male and a female prison in London (Pentonville and Holloway).

**Methods:**

Prisoners were sampled at random in a sequential procedure based on the Local Inmate Data System. We targeted equal numbers of male remand, male sentenced, female remand, and female sentenced prisoners. Following structured assessment of psychosis, common mental disorders, PTSD, personality disorders and disorders of abuse, we used the MRC Needs for Care Assessment (NFCAS) to establish whether potential needs for care in ten areas of mental health functioning were met, unmet, or incapable of being met by services.

**Results:**

Data on treatment experience were provided by 360 inmates. Eighty percent of females and 70% of males had at least one need for treatment. Over half (53.7%) of the needs of female prisoners were met, but only one third (36.5%) in males. Needs for medication were unmet in 32% of cases, while those for psychological treatment were unmet in 51%.

**Conclusions:**

Unmet needs for mental health treatment and care were common in the two prisons. This has adverse consequences both for individual prisoners and for the effective functioning of the criminal justice system.

## Introduction

In our companion paper [1], we reported very high rates of psychiatric disorder in two London prisons. Fifteen years previously the 1997 British National Survey of Psychiatric Morbidity among Prisoners identified similar rates of disorder, and also severe problems in delivering psychiatric treatment to prisoners [2]. Thus, prisoners often asked for help with their problems, but were twice as likely to have had such requests turned down after entering prison than in the period immediately beforehand, indicating that prison-based services were performing poorly [3, 4]. Provisions for rectifying this were set out in the late 1990s [5, 6]. The key principle was equity: prisoners should receive the same level of mental health care in prisons as they would in the wider community. To facilitate this, the National Health Service (NHS) was given responsibility for providing prison health care services in April 2006.

It was envisaged that prison mental health services would be more effective if locally commissioned [7]. Severe mental illness was to be managed by teams equivalent to NHS Community Mental Health Teams. Although around 90 in-reach mental health teams were subsequently commissioned in prisons, problems in implementing effective services were reflected in considerable local inequalities in mental health spending [8].

Brooker and Gojkovic [9] obtained data from 53 English prison in-reach teams. Although deploying on average only five whole time staff equivalents, most in-reach teams covered more than one prison. The workload was considerable: 75% of teams took more than 50 referrals a month, and staff complained that face-to-face interaction with prisoners was very restricted. They found it hard to deal with the complex problems associated with prisoners’ mental disorders, not helped by the lack of staff and expertise in prison primary care services.

The relationship between clients and medical services are traditionally subsumed under the concepts of demand, need, and utilisation. These concepts are distinct but related [10]. *Demand* is the requirement for services and treatments as seen by clients, and is based on lay knowledge, and lay concepts of disorder and treatment. Such concepts inevitably vary in their degree of sophistication, and are related to people’s individual illness perceptions. *Need* is the requirement for services and treatments identified from the professional (“expert”) perspective. It presupposes the identification of problems for which there are potentially effective interventions [10, 11]. It does imply a constructive and respectful interaction between clinicians and clients, and will often tally closely with the demand perspective. *Utilisation* is the actual take-up of services and treatments, and is shaped by practical issues such as the availability of services and the relative cost and effectiveness of treatments. However, it is also affected by the attitude of people to their health, and by the real or perceived accessibility of services. Inadequate treatment may result variously from faulty recognition of requirements by clients and service providers, and inadequate provision of treatment resources.

While UK prison mental health services have improved, investment must be guided by a clear account of the actual treatment needs of prisoners and their overall scale. Studies of the prevalence of psychiatric disorders in prisons give an idea of the burden of disorder. However, prevalence is merely a count of diagnosed cases, and there are problems in using it to determine service provision. It can only *suggest* the sorts of treatment and services required. It is thus unclear how the observed rates of mental illness in prison translate into specific requirements for treatment, or how the NHS services now responsible for their care should be configured. Prevalence information can be augmented by direct assessments of individual need [10, 12, 13], as clinicians quite properly do not base either their decisions to offer treatment or their choice of particular treatments purely on diagnosis; they take account of the way symptoms have evolved, how long they have lasted, the associated distress and impairment of social performance, and the possibility that symptoms will resolve quickly without treatment. Moreover, clients’ views must also be taken into account. However, clinician-defined needs assessments have an appreciable potential for idiosyncrasy, being dependent on individual clinical values that are often strongly held. An assessment of needs for treatment for research purposes therefore requires the standardisation of procedures for applying clinical judgements to need.

The purpose of the Assessing Needs for Psychiatric Treatment in Prisoners (ANPTP) project was (1) to quantify overall levels of the need for mental health care and treatment in prisoners, (2) to identify specific conditions requiring treatment, and (3) to assess how far these needs were met by the various mental health facilities in prison. To do this, we used a procedure that operationalises judgements of need, the Needs for Care Assessment (NFCAS [10, 13]). We chose this because it had been used in general population surveys [14, 15], and in preference to the forensic version of the Camberwell Assessment of Need [16], which in our view tends too much to conflate need and demand.

Our research was based in two prisons dealing with local remanded and sentenced prisoners, in which the responsibility for psychiatric services lay with local NHS Trusts. HMP Holloway was a female prison with an operational capacity of 512, while HMP Pentonville accommodated around 1200 male prisoners. Psychiatric services were reasonably well-organised, albeit provided by a range of agencies. Mood disorders were generally the province of primary care, with a process of triage governing referral to Community Mental Health In-reach Teams. Cases of psychosis were generally assessed and managed by the In-reach Teams. Psychological treatment and counselling were shared between voluntary providers and the prison psychology service. There were specific provisions for drug and alcohol problems, including detoxification regimes, and group and individual programmes of treatment. However, considerable staff turnover meant specific services sometimes became unavailable.

## Methods

Prisoners were randomly sampled in equal numbers from the following groups: male remand; female remand; male sentenced; female sentenced. The sequential sampling procedure and its rationale are described in detail in the companion paper [1], as are the instruments for identifying psychiatric disorder. Participants were interviewed in a single phase.

### Instruments

We used the NFCAS [13] to establish how far psychiatric services were successful in identifying and meeting the needs for treatment of prisoners with psychiatric disorders, and to what extent prisoners were willing to accept such treatment. Treatments were considered in detail and included pharmacotherapy, and a range of different sorts of psychological treatments. This allows inferences about the demands on services if all needs were met. The NFCAS standardises the coverage of both disorders and treatments, and then links disorders with treatment through rules that define need operationally [10]. Explicit guidelines and examples are incorporated in a manual. In the prison setting, we used the Community Version, as inmates were ostensibly incarcerated from the community without regard to their mental health status [13, 17, 18]. This version was designed to approximate, in a more itemised and systematic manner, the functioning of well-organised primary care and psychiatric services. Its reliability was established by Lesage et al. [19].

The definition of a primary need for care requires two distinct criteria (1) the person’s functioning falls below, or threatens to fall below, some minimum specified level (in the community context, this means significant distress from symptoms, with or without disablement), and (2) this is potentially remediable or preventable. For each area of clinical functioning covered, the assessment specifies the threshold for identifying impaired functioning and a set of appropriate interventions or *items of care*. Needs for care in each area are then determined by comparing the actual items of care provided with a model of what those items of care should be, based on the literature on treatment efficacy, particularly where this forms the basis of contemporary UK national guidelines.

Unlike conventional measures of symptoms and behaviour, the assessment uses data on level of functioning to identify the appropriate actions to be taken by clinicians. Needs are defined in terms of these actions, i.e. the offer of specific items of care.

The primary need status in each area of functioning falls into the categories:

- met need: appropriate action is already being undertaken
- unmet need: there is some action appropriate now that has not been undertaken
- no need: there is no clinical problem requiring treatment
- no meetable need: there is disablement but no action that is both appropriate and feasible.

Where unmet needs were identified, clinical judgements were made of the significance of the consequences for the prisoner’s well-being (mild, moderate or severe).

As the basis for identifying need, the authors of the instrument stipulated how long symptoms must last before treatment should be considered necessary; they took as their threshold the presence of clinically significant (i.e. moderate or severe rather than mild) psychiatric symptoms or disability over a period of 6 weeks. We evaluated needs in relation to specific areas of functioning: *positive psychotic symptoms*; *depressive symptoms*; *anxiety and obsessional symptoms*; *adjustment disorder clearly secondary to an external event or circumstance*; *posttraumatic stress disorder*; *personality disorder; problems with alcohol*; and *problems with drugs* (we did assess eating disorders, but these, perhaps surprisingly, were identified only in three women prisoners). While actual services differ considerably in resource and philosophy of care, this is deliberately not taken into account. To compare services, unmet needs in a given service are rated without considering whether particular items of care are routinely provided, or whether the staffing and expertise exists to provide them.

In this study, a panel of clinical assessors made consensus judgements of treatment needs on the basis of presentations of the available information by SJ. The panel included PB and NM, then the clinical leads for prison mental health care in HMP Holloway and HMP Pentonville, together with other members of the research team when available).

The NFCAS requires information about mental state and the course of disorder, social functioning, social stresses, the treatment received and the service users’ attitudes towards them. These requirements were met by a range of instruments.

Where possible and appropriate we used the same instruments for defining aspects of the mental state as the ONS Prisons Survey [2], as described fully in the companion paper [1]. They included the *Revised Version of the Clinical Interview Schedule* (CIS-R) [20] to assess neurotic symptoms and common mental disorders. We used information from the *Life Events and Difficulties Schedule* [21] to decide where affective symptoms should be interpreted as adjustment disorder. Psychotic disorders were assessed using SCAN [22, 23]. We also used the *Structured Clinical Interview for DSM*-IV [24] to identify personality disorder, and the *Posttraumatic Stress Diagnostic Scale* (PDS [25]). Alcohol misuse and dependence was assessed from the *Alcohol Use Disorders Identification Test* (AUDIT [26]) and the *Severity of Alcohol Dependence Questionnaire* (SAD-Q [27]). Drug dependence was identified from the questions in the ONS prison survey [2].

*The Social and Occupational Functioning Assessment Scale* (*SOFAS*) [28] is a simple measure of social functioning rated without consideration of the level of mental disturbance.

Finally, we used a structured interview to collect detailed information about potential and actual psychiatric treatments, and participants’ views about treatments offered or appropriate for their psychiatric symptoms. Their views are important as they are grounds for discriminating between unmet and unmeetable need: where participants reject treatment either specifically or as a general principle, ostensible needs must then be rated as unmeetable.

In the current report, we present straightforward cross-tabulations of need status in male and female and in remanded and sentenced prisoners with Chi-square tests of significance.

## Results

Ten sentenced and ten remand prisoners were sampled per month. The sampling procedure is described in the companion paper [1]. We interviewed 197 male and 171 female prisoners with an overall response rate of 70%. Most failures were due to unpredicted unavailability, rather than to refusal, and we were unable to obtain information on nonresponders with any consistency. We also failed to collect information about treatment for five male and three female interviewed prisoners. Sociodemographic characteristics are described in the companion paper [1].

Seventy-five percent of prisoners had at least one clinical condition for which treatment should have been considered, somewhat more so in women than in men (Table 1). In a minority, identified disorders were in abeyance, but with a significant risk of recurrence requiring continuing treatment and surveillance. In particular, alcohol and drug abuse is (almost totally) constrained in prison, and intervention is therefore aimed at minimising the risk of resumption on discharge.

**Table 1:** Prevalence of significant clinical problem by sex and sentencing category

Problem	Male (%)	Female (%)	Total (%)	Remand (%)	Sentenced (%)	Total (%)
Psychosis	22 (11.5)	9 (5.4)*	31 (8.6)	21 (12.)^+^	10 (5.9)	31 (8.6)
Depression	65 (33.9)	76 (45.2)*	141 (39.2)	73 (42.2)	68 (36.7)	141 (39.2)
Anxiety	17 (8.5)	12 (7.1)	29 (8.1)	18 (10.4)	11 (5.9)	29 (8)
Adjustment disorder	8 (4.2)	11 (6.5)	19 (5.3)	9 (5.2)	10 (5.3)	19 (5.3)
PTSD	9 (4.7)	20 (11.9)*	29 (8.1)	14 (8.1)	15 (8)	29 (8)
Personality disorder	31 (16.1)	33 (19.6)	64 (17.8)	32 (18.5)	32 (17)	64 (17.2)
Alcohol abuse	75 (39.1)	74 (44)	149 (41.4)	76 (43.9)	73 (38.2)	149 (41.3)
Substance abuse	79 (41.1)	86 (51.2)	165 (45.8)	91 (52.6)^+^	74 (39.4)	165 (45.7)

Male vs female: * *p* < 0.05; remand vs sentenced: ^+^ *p* < 0.05

The overall prevalence of psychosis was particularly striking. It was significantly more common in remanded than in sentenced, and in female than in male prisoners. However, depression was a more prevalent clinical problem, being present in around 34% of male and 45% of female prisoners. Rates for anxiety as a *clinically significant* problem were lower. This is because, in many prisoners, anxiety symptoms were relatively mild and not very disturbing or disabling, while in others the clinical picture was dominated by depressive symptoms, so we subsumed the anxiety symptoms under the rubric of depression. This is reflected in the concurrence of these two disorders: 16% of prisoners with depression also had a separate anxiety disorder, whereas three-quarters of cases of anxiety also had depression. In other instances, relatively mild depressive or anxious symptoms could be wholly attributed to the difficulties of adjusting to the fact of imprisonment and the demands of prison life. Where such symptoms were persistent, this generally led to their being recorded as an adjustment disorder.

Overall, 5% of prisoners were deemed to have adjustment disorders, sometimes related to the stresses attendant on imprisonment, sometimes to ongoing situations outside the prison. This low rate is the result of the NFCAS criterion that, to be registered, disorders must be present for 6 weeks. PTSD is distinct from adjustment disorder, both by the extreme nature of the stressor involved and by its specific symptoms. Eight percent of prisoners were currently suffering from posttraumatic stress disorder. Depression, anxiety, adjustment disorder, and PTSD comprise a general category of affective disorders. Forty-nine percent of prisoners suffered from affective disorder so defined (55.6% of women, 43.2% of men).

The identification of personality disorder in prisoners is problematic, particularly as the inclusion criteria for antisocial personality disorder include criminal activity and the responses to it of the criminal justice system. In the NFCAS, we identified personality disorder on the basis of additional characteristics for which it would be appropriate to consider treatment. As recorded in Table 1, this resulted in prevalence lower than in the National Prisoners Survey, generally because criminality of itself did not necessarily indicate to us a need for psychiatric treatment [29]. Thus, we identified around 18% of male and female prisoners as having personality disorder meriting a consideration of treatment.

The really striking feature in Table 1 is the very high prevalence of problems with alcohol and drug abuse. Overall, 41% of prisoners had problems with alcohol, and 46% with drug abuse. These problems were often linked: of the 225 prisoners with one or other of these conditions, 89 (39.6%) had both.

Depression, alcohol abuse and drug abuse are likely to reinforce each other, and there was considerable overlap: of the 225 prisoners who had one of these conditions, 51 (22.7%) had all three. Of 141 prisoners with depression, 105 (73.9%) had at least one abuse disorder, whereas fewer than half those who abused alcohol or drugs were depressed.

In Table 2 we present the need and treatment status of the various disorders. In people with psychotic conditions, about half of all needs were met, and about a tenth could not be met because of external constraints, mainly the refusal of prisoners to countenance treatment. However, a need for some kind of treatment identified as appropriate remained unmet in 40% of prisoners with psychotic problems. Levels of unmet need were greater in male than in female prisoners, and in remand than in sentenced prisoners, though not significantly so. Antipsychotic medication is of course a mainstay of treatment in psychosis, and a failure, at least to consider it, is reproachable. While three prisoners explicitly rejected offers of medication, around a quarter had an unmet need for it, predominantly where their condition had gone unrecognised. In two female cases, the prescription of medication was rated as an *overprovision,* since their symptoms had abated more than a year ago and their medication had not been reviewed.

**Table 2:** Overall need status for treatment of different disorders

Disorder/need status	Male	Female	Remand	Sentenced	Total
**Psychosis** (*N*)	22	8	11	19	30
Met (%)	10 (45.5)	5 (62.5)	4 (36.4)	11 (57.9)	15 (50)
Unmet (%)	10 (45.5)	2 (25)	5 (45.5)	7 (36.8)	12 (40)
Unmeetable (%)	2 (9.0)	1 (12.5)	2 (18.1)	1 (5.3)	3 (10)
**Depression** (*N*)	50	63	55	58	113
Met (%)	10 (20)	32 (50.8)**	21 (38.2)	21 (36.2)	42 (37.2)
Unmet (%)	37 (74)	28 (44.4)	30 (54.5)	35 (60.3)	65 (57.5)
Unmeetable (%)	3 (6)	3 (4.8)	4 (7.3)	2 (3.4)	6 (5.3)
**Anxiety** (*N*)	8	3	4	7	11
Met (%)	2 (25)	1 (33.3)	2 (50)	1 (14.3)	3 (27.2)
Unmet (%)	6 (75)	1 (33.3)	2 (50)	5 (71.4)	7 (63.7)
Unmeetable (%)	0	1 (33.3)	0	1 (14.3)	1 (9.1)
**Adjustment disorder** (*N*)	6	6	6	6	12
Met (%)	0	2 (33.3)	1 (16.7)	1 (16.7)	2 (16.7)
Unmet (%)	5 (83.3)	4 (66.7)	5 (83.3)	4 (66.6)	9 (75)
Unmeetable (%)	1 (16.7)	0	0	1 (16.7)	1 (8.3)
**PTSD** (*N*)	5	14	10	9	19
Met (%)	1 (20)	1 (7.1)	1 (10)	1 (11.1)	2 (10.5)
Unmet (%)	4 (80 %)	13 (92.9 %)	9 (90 %)	8 (88.9 %)	17 (89.5 %)
Unmeetable (%)	0	0	0	0	0
**Personality disorder** (*N*)	16	19	21	14	35
Met (%)	4 (25)	7 (36.8)	7 (33.3)	4 (28.6)	11 (31.4)
Unmet (%)	11 (68.7)	11 (57.9)	13 (61.9)	9 (64.3)	22 (62.9)
Unmeetable (%)	1 (6.3)	1 (5.3)	1 (4.8)	1 (7.1)	2 (5.7)
**Alcohol abuse** (*N*)	57	66	55	68	123
Met (%)	20 (35.1)	38 (57.6)*	27 (49.1)	31 (45.6)	58 (47.2)
Unmet (%)	28 (49.1)	19 (28.8)	23 (41.8)	24 (35.3)	47 (38.2)
Unmeetable (%)	9 (15.8)	9 (13.6)	5 (9.1)	13 (19.1)	18 (14.6)
**Drug abuse** (*N*)	65	75	61	79	140
Met (%)	35 (53.8)	48 (64)	32 (52.5)	51 (64.6)	83 (59.3)
Unmet (%)	24 (36.9)	16 (21.3)	19 (31.1)	21 (26.6)	40 (28.6)
Unmeetable (%)	6 (9.3)	11 (14.7)	10 (16.4)	7 (8.8)	17 (12.1)

Male vs female: * *p* < 0.05; ** *p* < 0.01

Just over a third of prisoners with depressive conditions had their needs for treatment met, nearly 60% did not. However, there was a marked sex difference: needs for treatment was unmet in three-quarters of male prisoners. This was unlikely to be due to a more limited availability of treatment in HMP Pentonville. A more plausible explanation lies in a tendency for male prisoners to acknowledge depressive symptoms less readily, together with a relative failure of surveillance of such symptoms in the men’s prison. This finding may also be partly explained by the fact that one third of prisoners had been in Pentonville for under a month. Many prisoners experienced low mood during their first few weeks in prison and it is not a straightforward matter for prison staff to identify those needing referral to primary care for treatment. Even when an appointment to see a GP had been arranged, prisoners would often be moved to another prison before the assessment.

Anxiety disorders were often comorbid with other conditions, and this required a judgement about which condition was the primary and appropriate target of treatment. Nevertheless, the situation regarding treatments for anxiety was similar to that seen in depressive disorders, with many unmet needs.

The relative mildness of identified adjustment disorders may be reflected in the fact that most needs for treatment went unmet, probably because they were overlooked.

In all, 19 prisoners (8.1%) were assessed as requiring treatment for PTSD (Table 2). In two further cases, the need for treatment for PTSD was subsumed under treatment for a different clinical problem. The identification and treatment of this disorder was poor in both prisons: 90% of identified needs were unmet.

Historically, psychiatrists and clinical psychologists have been pessimistic about the treatability of personality disorder. However, many people diagnosed with personality disorders are survivors of abuse, particularly in prisons. There are known ways of dealing with both the emotional and the behavioural consequences of abuse, primarily involving psychological techniques. We used the Needs for Care Assessment to judge whether individual prisoners might respond to such interventions if offered. In two thirds of both male and female cases needs went unmet. Interestingly, these prisoners were relatively rarely rated as having unmeetable need—in other words they were amenable in principle to being treated (Table 2).

Both prisons in this study were committed to offering interventions to prisoners with alcohol problems, particularly in the form of individual and group psychological treatments. One in seven of identified needs were rated as unmeetable, usually due to prisoners’ reluctance to accept treatment. There was no gender difference in unmeetable need. However, a majority of meetable needs went unmet in male prisoners, whereas they were largely met in women.

Drug abuse was the most prevalent condition in these prisoners. Again, the prisons have well developed systems and interventions to help inmates with such problems, and nearly 60% of needs were met. Once more, an appreciable number of prisoners declined involvement in what was judged appropriate treatment.

Table 3 records our evaluation of the overall delivery of appropriate treatments. Taking all treatments together, around 10% of needs were unmeetable, while 44.7% were met and 45.3% unmet. Women’s treatment needs were significantly more likely to be met than those of men. Specific needs for medication were more likely to be met (59%), and again significantly more so in women. The deployment of psychological treatments, broadly conceived, was almost as good, with half the need being met, again more so in women. However, many of the psychological treatments comprised counselling, delivered through the relatively well-organised services for drug and alcohol problems. More than half the psychological treatment needs for these disorders were met, in contrast to fewer than 40% for other disorders (Table 4).

**Table 3:** Overall success in meeting treatment needs

Treatment	Male	Female	Remand	Sentenced	Total
**All treatments** (*N*)	229	254	223	260	483
Met (%)	82 (35.8)	134 (52.8)**	95 (42.6)	121 (46.5)	216 (44.7)
Unmet (%)	125 (54.6)	94 (37.0)	106 (47.5)	113 (43.5)	219 (45.3)
Unmeetable (%)	22 (9.6)	26 (10.2)	22 (9.9)	26 (10.0)	48 (9.9)
**Medication** (*N*)	47	55	56	46	102
Met (%)	17 (36.2)	43 (78.2)**	35 (62.5)	25 (54.3)	60 (58.8)
Unmet (%)	23 (48.9)	10 (18.2)	16 (28.6)	17 (37.0)	33 (32.4)
Unmeetable (%)	7 (14.9)	2 (3.6)	5 (8.9)	4 (8.7)	9 (8.8)
**Psychological treatments** (*N*)	125	189	153	161	314
Met (%)	54 (43.2)	100 (52.9)	77 (50.3)	77 (47.8)	154 (49.0)
Unmet (%)	52 (41.6)	66 (34.9)	54 (35.3)	64 (39.8)	118 (37.6)
Unmeetable (%)	19 (15.2)	23 (12.2)	22 (14.4)	20 (12.4)	42 (13.4)

Male vs female: ** *p* < 0.01

**Table 4:** Psychological treatment needs and disorder categories

Disorder categories	Male	Female	Remand	Sentenced	Total
**All disorders** (*N*)	125	189	153	161	314
Met (%)	54 (43.2)	100 (52.9)	77 (50.3)	77 (47.8)	154 (49.0)
Unmet (%)	52 (41.6)	66 (34.9)	54 (35.3)	64 (39.8)	118 (37.6)
Unmeetable (%)	19 (15.2)	23 (12.2)	22 (14.4)	20 (12.4)	42 (13.4)
**Alcohol and drug abuse only** (*N*)	68	108	91	85	176
Met (%)	33 (48.5)	66 (61.1)	55 (60.4)	44 (51.8)	99 (56.3)
Unmet (%)	22 (32.4)	25 (23.1)	20 (22.0)	27 (31.8)	47 (26.7)
Unmeetable (%)	13 (19.1)	17 (15.7)	16 (17.6)	14 (16.5)	30 (17.0)
**Excluding alcohol and drug abuse** (*N*)	57	81	62	76	138
Met (%)	21 (36.8)	34 (42.0)	22 (35.5)	33 (43.4)	55 (39.9)
Unmet (%)	30 (52.6)	41 (50.6)	34 (54.8)	37 (48.7)	71 (51.4)
Unmeetable (%)	6 (10.5)	6 (7.4)	6 (9.7)	6 (7.9)	12 (8.7)

**Fig. 1:**
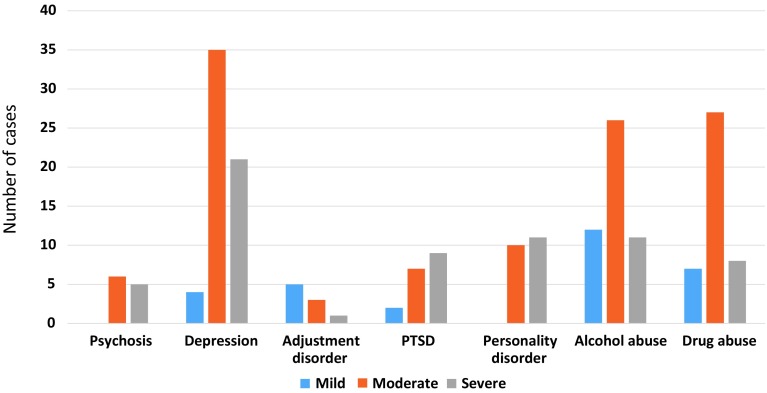
The significance of unmet need

Figure 1 presents our judgements of the consequences of unmet needs for the well-being of the prisoner. These were more severe in cases of depression, psychosis, personality disorder, and PTSD. These data indicate where increased effort on the part of prison mental health services might yield the greatest numerical or individual benefit. The utilitarian view would advise a focus on depression, alcohol and substance abuse, while equity would argue redoubled effort to treat psychosis, personality disorder and PTSD.

## Discussion

In the companion paper, we reported very high prevalence rates for mental disorders using standardised diagnostic instruments [1]. The procedures used here were somewhat different. We combined diagnostic and other clinical information to identify people for whom some form of treatment should be considered. In general, this would reduce prevalence by excluding relatively mild disorders likely to be self-limiting. The existing literature about the prevalence of mental disorders in prison can therefore be no more than a frame of reference for our specific findings [30]. Nevertheless, we found a majority of prisoners had mental disorders significantly affecting their behaviour, functioning and well-being, for which treatment ought therefore to be considered. The disorders with the highest prevalence rates were depression, alcohol problems and substance abuse. The high prevalence of psychotic disorders identified here as having needs for treatment are commensurate with the SCAN-based diagnoses in the companion paper, where the issue of prevalence is discussed. These disorders were not substance-related.

Comorbidity was also very frequent: this creates particular difficulties for the effective provision and deployment of mental health services in prisons, especially if the disorders are of different types (e.g. a mental disorder in tandem with substance abuse problems). In contrast, interventions outside the prison environment will capture relatively few people with multiple disorders, even though comorbidity will affect responses to treatment [31].

Over 80% of female and 70% of male prisoners were identified as needing treatment for a psychiatric condition. Our results do show that efforts were being made in both prisons to identify people in need, and to offer them treatment. Nevertheless, of meetable needs, half were met and half unmet, a significant failure of recognition somewhere in the process of assessment. Assessment operates through a series of filters (recognition by reception staff, by officers working in the body of the prison, and by more specialist staff of one sort or another). Thus, the identification of need arises from the interaction between staff and prisoners, and can fail for a variety of reasons: staff may not ask, and prisoners may not tell.

Only 10% of needs were judged unmeetable: it was four times more likely that treatment was not offered than that it would be declined. Needs for medication were met more often than needs for psychological treatments (around 70% compared with 50%). However, the delivery of psychological treatments was inflated by the counselling afforded in the drug and alcohol programmes. Otherwise, only 40% of psychological treatment needs were met. Indeed, other than in the drug and alcohol programmes there was virtually no provision for psychological treatment in Pentonville prison during the course of this study. Indeed male prisoners fared worse than females in several ways. Depression was often unrecognised in male prisoners. Consequently, even the relatively straightforward prescription of medication was only provided in a third of appropriate cases. While half the treatment needs for alcohol problems were met overall, women prisoners were again better served. Treatment of substance abuse was generally better than for alcohol problems, and it was only here that the provision for men approached in quality that experienced by women prisoners.

Rating that a need has been met does not of course guarantee the intervention will be successful, only that it is a rational attempt to deal with a clinical problem in the context of our current knowledge of effectiveness. In the absence of anything better, even an unreliably effective treatment is a rational choice.

It should be noted that similar failures also characterize treatment provision in the general population [14, 15], although the situation may have improved over the last 20 years [32]. However, disorders in prison are more severe [1, 2], and for many in our study the consequences of unmet need appeared serious. Prison mental health services remain under-resourced [9], prison regimes do not conduce to effective treatment delivery, and incarceration disrupts treatment planning. Diversion schemes need to be maximised so imprisonment is avoided where possible. This requires the active and effective cooperation of community mental health trusts. These requirements have been acknowledged for 20 years but without effective implementation of policy.

### Strengths and limitations

The study involves a large and representative sample of prisoners from two general prisons in London. We used standard diagnostic instruments, and an intensive technique for the assessment of needs and the extent to which they had been met. This has never been done before in a prison setting. Our findings are striking, and we have no reason to expect that treatment is delivered more effectively in other, similar prisons [9]. We inevitably missed a proportion of prisoners who were discharged or moved quickly; this places constraints on our findings, particularly if they were diverted specifically for mental health reasons.

## Conclusions

We have documented particular and serious problems with the psychiatric and psychological treatment of people in prisons. The limited availability of suitable placements often seriously disrupted the necessary continuity of care. Without warning, prisoners might be released from courts or transferred to other prisons, which was very disruptive to after-care arrangements. Many prisoners lack a settled destination on release [33], and few can access the stabilising effects of employment. Shorter periods of imprisonment are particularly likely to cause problems with mental health treatment as they impair access to mental health services in prison. The prisoner’s community tenure has consequently been disrupted with no compensatory opportunity for improvements to their mental health. This should be acknowledged in considerations of sentencing policy.

In each case we estimated the significance of unmet need. This gives an idea of the impact of any future improvement in services. These will require the modification of demand, the recognition of need, and the extension of resource. Effort put into the treatment of depression, and alcohol and substance abuse services would be valuable because, although the unmet need was not judged important in every case, the prevalence of these conditions was high, and this is therefore where most cases of significant unmet need were located. On the other hand, much of the unmet need for psychosis, PTSD and personality disorder was regarded as having major consequences for the individual concerned.
